# Impact of Family and Friends on Antisocial Adolescent Behavior: The Mediating Role of Impulsivity and Empathy

**DOI:** 10.3389/fpsyg.2019.02071

**Published:** 2019-09-10

**Authors:** David Álvarez-García, Paloma González-Castro, José Carlos Núñez, Celestino Rodríguez, Rebeca Cerezo

**Affiliations:** ^1^Department of Psychology, University of Oviedo, Oviedo, Spain; ^2^Universidad Politécnica y Artística del Paraguay, Asunción, Paraguay

**Keywords:** family, friends, antisocial behavior, impulsivity, empathy, adolescence

## Abstract

Adolescence is an especially risky phase for the appearance of antisocial behaviors. Antisocial behavior produces significant individual and social harms, so it is important to provide keys for prevention and treatment. To do that, it is essential to identify the main predictors. The objective of this study is to analyze the effect of family (affection and communication, behavioral control) and friends (antisocial friendships) on adolescent antisocial behavior, as well as the mediating role of adolescent impulsivity and empathy on these relationships. Previously validated questionnaires measuring parental affection and communication, and behavioral control, as well as adolescent impulsivity, empathy, antisocial friendships, and antisocial behavior were applied to 3199 adolescents in Asturias (Spain), aged between 11 and 18 (M = 14.03; SD = 1.39). Descriptive, correlational, and structural equation analysis were performed. Antisocial friendships were a risk factor for antisocial behavior in adolescence, with a moderate effect size. The effect is mostly direct, although it is also indirect through the positive relationship with adolescent impulsivity and low empathy. The two analyzed parenting style dimensions (affection and communication, and behavioral control) demonstrate a protective effect, albeit small, on adolescent antisocial behavior. There is a direct protective effect, but it is mostly indirect through the negative relationship with antisocial friendships and low adolescent empathy. Parental behavioral control can be a risk factor for antisocial behavior, through the positive relationship with adolescent impulsivity. This study helps to clarify the causal mechanisms of antisocial behavior in adolescence, as a basis for its prevention and treatment.

## Introduction

Antisocial behavior is usually understood to be behavior that violates social norms and harms the rights of others (Peña and Graña, 2006). Many people exhibit this kind of behavior at some point in their lives. However, this is usually infrequent and limited to certain points in time and specific contexts. Adolescence is particularly prone to it, as it is a stage of seeking and experimentation which is key to the formation of personal identity and in which peer acceptance is particularly important. Antisocial behavior is occasionally used by adolescents to be accepted, or to improve their status within a group. Despite that, in a small number of cases, antisocial behavior can be a stable characteristic that persist into adulthood (Moffitt, 1993).

Antisocial behavior produces significant personal and social harm. A person who engages in this behavior, especially if sustained over time, may have reduced educational or work opportunities; it may lead to maladjusted behaviors in adulthood (substance abuse, criminal activities), as well as mental health issues; and it might lead to legal consequences. Those affected by this behavior may suffer physical, emotional or economic consequences. The social consequences of this behavior consume significant resources in mental health, education and juvenile justice systems (Cook et al., 2015; Sawyer et al., 2015).

Antisocial behavior in adolescence, and its persistence into adulthood, has been explained by the interaction between personality traits of vulnerability and environmental factors which strengthen or inhibit these traits. Certain personality traits constitute a vulnerability for the development of antisocial behavior depending on contextual factors. In order to prevent the problem it is necessary to identify these variables, which act as protective or risk factors (Álvarez-García et al., 2018). Regarding environmental factors, the most important contexts of influence and socialization for adolescents are family and friends. However, there is little current research with large samples that looks at how impulsivity and empathy specifically modulate the effect of family and friends on antisocial behavior in adolescence.

Parenting practices influence children’s behavior (Ruiz-Hernández et al., 2019). The different parenting styles have traditionally been defined in terms of two dimensions: responsiveness and demandingness (Maccoby and Martin, 1983). Close relationships, support and communication on the part of parents, as well as setting limits of behavior and supervision of what their children do or experience, depending on their level of autonomy, are positive for child development. Parental warmth and behavioral control are protective factors for externalizing behaviors, aggression, delinquency, and consumption of alcohol or addictive substances (Hoskins, 2014), especially when they occur together. Both overprotection and hostile and intrusive control are risk factors for these problems (Pinquart, 2017).

In adolescence, the influence of family tends to decrease as the influence of friends increases. Antisocial friendships are a significant risk factor for both violent and non-violent antisocial behavior (Cutrín et al., 2017). The type of friendships can enhance or diminish the influence of parenting practices (Lansford et al., 2003).

Parents can influence their children’s friendships, directly or indirectly. For example, parent’s decisions can affect the type of friends their children will have (choice of neighborhood, school, activities, etc.); they can transmit attitudes, values and abilities which mold and influence their children’s behavior, social reputation, and groups they join (Brown et al., 1993). Parental monitoring is a protective factor for deviant peers (Cutrín et al., 2015). In addition, if parents establish a warm, communicative environment, it is more likely for the adolescents to spontaneously tell them about what is happening in their lives, what they do, and who they do it with (Álvarez-García et al., 2016a), which makes it easier for the parents to exercise some control over their children’s friendships.

These two contexts (family and friends) can not only influence adolescents’ antisocial behavior directly, but also indirectly through their effect on certain personality traits such as impulsivity and a lack of empathy, which increase the likelihood of behaving in an antisocial manner. Impulsivity refers to having difficulties in controlling impulses, acting without considering the consequences of the action for oneself or others (Stahl et al., 2014). Previous research has found higher levels of self-control in children in families with a parenting style characterized by affection and communication (Wills et al., 2004), behavioral control (Li et al., 2015) and authoritative parenting style (Burt et al., 2006). The positive effects of parental control occur mainly when it happens in a warm parental context. In addition, there is evidence that deviant peer relationships are a risk factor for impulsivity (Burt et al., 2006). Impulsivity is in turn a risk factor for antisocial behavior (Jones et al., 2011).

Empathy is usually defined as the capacity to understand and share others’ feelings (Oliveira et al., 2018). There is ample evidence that a lack of empathy is a significant risk factor for antisocial behavior (Van Langen et al., 2014). It is not only a risk factor for antisocial behavior which harms others. Behavior which harms oneself, such as the consumption of illegal drugs, may be more likely in those with low empathy. They may have difficulties identifying significant others’ disapproval (concern, anger, fear, etc.) and the negative consequences of their behavior on others (Massey et al., 2018). In a similar way to self-control, empathy is positively related to parental warmth (Boele et al., 2019), parental control (Asano et al., 2016) and authoritative parenting style (Mesurado and Richaud, 2017). There is also evidence of a negative association between relating with antisocial friendships and empathy (Padilla-Walker and Bean, 2009).

**FIGURE 1:**
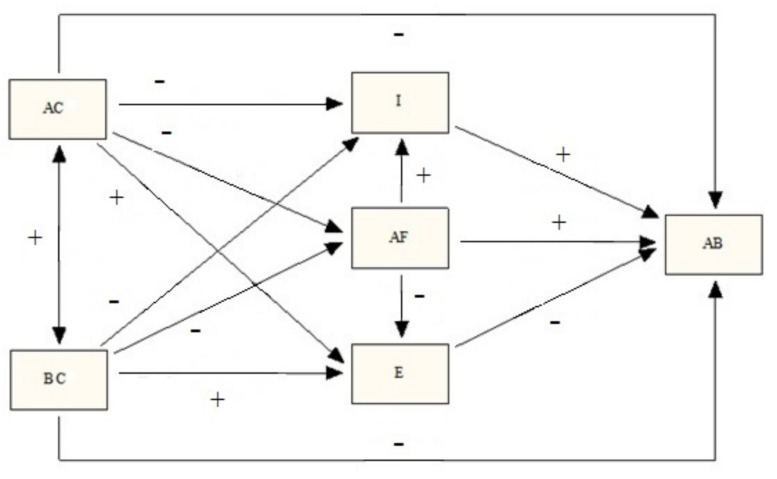
Starting theoretical model (AC, affection and communication; BC, behavioral control; I, impulsivity; AF, antisocial friendships; E, empathy; AB, antisocial behavior; +, positive relation; −, negative relation).

The aim of this work is to analyze the effect of family (affection and communication, behavioral control) and friends (antisocial friendships) on adolescent antisocial behavior, and the mediating role of adolescents’ impulsivity and empathy, in a large sample of Spanish adolescents. Given the research examined previously, we expect the theoretical model shown in Figure 1 to have a good fit to the empirical data.

## Materials and Methods

### Participants

We selected 20 schools from all the publicly funded schools providing compulsory secondary education in Asturias (Spain) via stratified random sampling with clusters. The population of schools was divided in two groups according to type (public or private-concerted) and a number of schools in each group proportional to the population were randomly selected. The questionnaires were given to all students in the 4 years of compulsory secondary education in each school, a total of 3360 students.

We selected those students for the study who had reported living with one or both parents, and eliminated those with a significant number of unanswered questions or void responses. The final sample comprised 3199 students, aged between 11 and 18 (M = 14.03; SD = 1.39), of which 49.2% were girls. The final sample constitutes the 95.21% of the total of students who responded the questionnaires.

### Measures

#### Affection and Communication

The perceptions of adolescents regarding their parents’ support, emotional closeness, and willingness to talk were assessed by the “affection and communication” factor from the *Dimensions of Parenting Style Questionnaire* (Álvarez-García et al., 2016b), an adaptation of the scale developed by Oliva et al. (2007). It consists of four items (“Cuando hablo con mis padres, muestran interés y atención” [“When I talk to my parents, they show interest and pay attention”], “Mis padres me animan a que les cuente mis problemas y preocupaciones” [“My parents encourage me to tell them my problems and concerns”], “Si tengo algún problema puedo contar con la ayuda de mis padres” [“If I have a problem, I can count on my parents’ help”], and “Mis padres muestran interés por mí cuando estoy triste y enfadado/a” [“My parents show interest in me when I am sad and angry”]), with four response options (from 1, completely false, to 4, completely true). The total score for each respondent in this factor corresponds to the sum of the scores on each item (minimum 4, maximum 16). High scores indicate high levels of affection and communication. The internal consistency of this scale in this study sample is high (α = 0.87).

#### Behavioral Control

To measure parental control of activities as perceived by adolescents, the “behavioral control” factor from the *Dimensions of Parenting Style Questionnaire* by Álvarez-García et al. (2016b) was used. It is an adaptation of the scale developed by Oliva et al. (2007). It consists of four items (“Mis padres intentan saber a dónde voy cuando salgo” [“My parents try to know where I am going when I leave home”], “Si vuelvo tarde a casa, mis padres me preguntan por qué y con quién estuve” [“If I return home late, my parents ask me why and who I was with”], “Mis padres ponen límites a la hora a la que debo volver a casa” [“My parents set limits on the time that I should return home”], and “Mis padres me preguntan en qué gasto el dinero” [“My parents ask me how I spend money”]), in which respondents are asked to assess the extent to which each statement is true (from 1, completely false, to 4, completely true). The total score for each respondent in this factor corresponds to the sum of the scores on each item (minimum 4, maximum 16). High scores indicate high levels of behavioral control. The internal consistency of this scale with this study sample is adequate (α = 0.75).

#### Impulsivity

The degree of the respondents’ impulsivity was assessed using a self-reported scale previously used by the research team (Álvarez-García et al., 2016a). The scale was created using part of the impulsivity criteria proposed by the DSM-5 for the diagnosis of Attention Deficit and Hyperactivity Disorder (American Psychiatric Association, 2013). It consists of five items: “En clase o en juegos, a menudo me cuesta esperar turno, por lo que me cuelo o interrumpo” [“In class or when playing games, it is often difficult for me to wait my turn, so I jump in or interrupt”], “A menudo contesto antes de que se haya completado la pregunta” [“I often answer before the question has finished”], “A menudo digo lo que me viene a la cabeza, sin pensar primero sus consecuencias o si es oportuno para la conversación” [“I often say what comes to mind without thin-king first of the consequences or whether it is appropriate for the conversation”], “A menudo hago cosas sin pensar en las consecuencias” [“I often do things without thinking of the consequences”] and “Habitualmente me resulta difícil esperar turno, por lo que me adelanto a hablar cuando no me corresponde o interrumpo a quien está hablando” [“Usually, I find it difficult to wait my turn, so I jump into speak when it is not my turn or I interrupt the person talking”] The response is a Likert-type scale with four options (from 1, completely false, to 4, completely true). The total score for each respondent in this factor corresponds to the sum of the scores on each item (minimum 5, maximum 20). High scores indicate high levels of impulsivity. The internal consistency of the scores obtained with the scale in this study sample is adequate (α = 0.76).

#### Empathy

The degree of empathy in the adolescents evaluated was measured by a self-reported scale previously used by the research team (Álvarez-García et al., 2016a). It is composed of five items that refer to the extent to which a respondent believes that he/she is capable of identifying with others and sharing his/her feelings: “Siento las desgracias de los demás” [“I feel the misfortunes of others”], “Si se burlan de un compañero, me siento mal pensando en lo mal que lo está pasando” [“If a classmate is teased, I feel bad thinking about what is happening to him/her”], “Soy paciente con las personas que hacen las cosas peor que yo” [“I am patient with people who do things worse than I do”], “Cuando veo que un/a amigo/a está triste, yo también me entristezco” [“When I see that a friend is sad, I also become sad”] and “Me alegro cuando le pasa algo bueno a un conocido” [“I am happywhen something good happens to someone I know”]. The response is a Likert-type scale with four options (from 1, completely false, to 4, completely true). The total score for each respondent in this factor corresponds to the sum of the scores on each item (minimum 5, maximum 20). High scores indicate high levels of empathy. The internal consistency of the scores obtained with the scale in this study sample is adequate (α = 0.67).

#### Antisocial Friendships

To assess the extent to which respondents relate to antisocial friendships, a scale previously used by the research team was used (Álvarez-García et al., 2016b). It comprises four items, in which the respondents indicate whether the situation described has occurred during the past year: “Alguno/a de mis mejores amigos/as ha ensuciado, dañado o destruido conscientemente mobiliario público (por ej., una pared, una papelera, una farola, asientos del autobús)” [“One or some of my best friends have soiled, damaged, or destroyed public furniture (e.g., a wall, a trashcan, a lamppost, seats on the bus)”], “Alguno/a de mis mejores amigos/as ha robado algo de una tienda, del colegio o de casa” [“One or some of my best friends have stolen something from a shop, school, or a private home”], “Alguno/a de mis mejores amigos/as se ha peleado físicamente en serio con otro/a chico/a” [“One or some of my best friends have had a real physical fight with another young person”], and “Alguno/a de mis mejores amigos/as ha consumido drogas ilegales” [“One or some of my best friends have consumed illegal drugs”]. The response requested from the respondent is dichotomous (1 = true, 0 = false). The total score for each respondent in this factor corresponds to the sum of the scores on each item (minimum 0, maximum 4). High scores indicate high levels of antisocial friendships. The internal consistency of the scale in this sample is adequate (KR20 = =0.710.71).

#### Antisocial Behavior

To assess the extent to which respondents recognize engaging in different types of antisocial behavior, a self reported scale was used. It consists of four items, referred to the same four types of antisocial behavior as the antisocial friendships questionnaire: “He ensuciado, dañado o destruido conscientemente mobiliario público (por ej., una pared, una papelera, una farola, asientos del autobús)” [“I consciously soiled, damaged, or destroyed public furniture (e.g., a wall, a trashcan, a lamppost, seats on the bus)”], “He robado algo de una tienda, del colegio o de una casa” [“I stole something from a shop, school, or a private home”], “He golpeado o me he peleado con un desconocido hasta dañarle” [“I have hit or fought with a stranger to the point of harming him/her”], and “He consumido drogas ilegales” [“I used illegal drugs”]. The response requested from respondents is dichotomous (1 = true, 0 = false), indicating whether they have performed these activities at least once in the last year. The total score for each respondent in this factor corresponds to the sum of the scores on each item (minimum 0, maximum 4). High scores indicate high levels of antisocial behavior. The internal consistency of the scale in this sample is adequate (KR20 = 0.65).

### Procedure

Permission to administer the questionnaires was requested from the head teacher in each selected school. Each school obtained family consent for the participation of the students in the study because they were underage. The questionnaires were completed by the students at the school during school hours. At the time of the application of the questionnaires, participants were informed of the voluntary and anonymous nature of the test as well as the confidential treatment of the data obtained.

### Data Analysis

The first step was to perform a descriptive analysis of the variables in the starting theoretical model (mean, standard deviation, response range, skewness and kurtosis). Following that, we calculated the correlation coefficients between each pair of variables. Given that the distribution of each variable was relatively close to normality (Kline, 2011), we used the Pearson correlation coefficient for that purpose. These preliminary analyses were performed using the statistical software SPSS 24 (IBM Corp, 2016b).

Following that, using AMOS 24 (IBM Corp, 2016a) statistical software, we used path analysis to assess how well the starting theoretical model fit the empirical data, as well as the magnitude of the direct and indirect effects of each variable. The method of estimation was the Maximum Likelihood method. To determine the degree of fit of the tested model, we used the Chi-square (χ^2^)/degrees of freedom (df) ratio, the Comparative Fit Index (CFI), the Standardized Root Mean Square Residual (SRMR), and the Root Mean Square Error of Approximation (RMSEA). The fit is usually considered good when CFI ≥ 0.95, SRMR ≤ 0.08, and RMSEA ≤ 0.06 (Hu and Bentler, 1999), and χ^2^/df < 3 (Ruiz et al., 2010).

## Results

### Preliminary Analysis

Although there was variation between the participants, the majority of they tended to report that their parents demonstrated affection and communication with them and controlled their behavior (setting limits and showing concern about what happened to them). They tended to describe themselves as empathic and having little impulsivity, little antisocial behavior and few antisocial friendships (Table 1). The distribution of scores in each variable was relatively close to normality.

**TABLE 1:** Descriptive statistics and Pearson correlation coefficients between the starting theoretical model variables.

	**1**	**2**	**3**	**4**	**5**	**6**
(1) Affection and communication						
(2) Behavioral control	0.30^∗∗∗^					
(3) Antisocial friendships	–0.20^∗∗∗^	–0.17^∗∗∗^				
(4) Impulsivity	–0.13^∗∗∗^	–0.03	0.34^∗∗∗^			
(5) Empathy	0.24^∗∗∗^	0.23^∗∗∗^	–0.17^∗∗∗^	–0.12^∗∗∗^		
(6) Antisocial behavior	–0.18^∗∗∗^	–0.18^∗∗∗^	0.59^∗∗∗^	0.28^∗∗∗^	–0.18^∗∗∗^	
Mean	13.89	13.39	1.24	10.25	15.23	0.43
SD	2.74	2.81	1.35	3.48	2.95	0.86
Response range	4–16	4–16	0–4	5–20	5–20	0–4
Skewness (SE = 0.04)	–1.55	–1.18	0.74	0.45	–0.67	2.19
Kurtosis (SE = 0.09)	2.02	0.95	–0.72	–0.38	0.52	4.41

*^∗∗∗^*p* < 0.001.*

Antisocial behavior was statistically significantly related to the other variables included in the starting model as predictor variables. The relationship is positive with antisocial friendships and impulsivity; and negative with affection and communication, parental control, and the adolescents’ empathy. All of the predictor variables were statistically significantly correlated with each other, except for behavioral control and impulsivity (Table 1).

### Path Analysis

The predictive model of antisocial behavior tested (Figure 1) demonstrated an adequate fit to the empirical data [χ^2^ = 12.820; df = 1; χ^2^/df = 12.820; CFI = 0.995; RMSEA = 0.061 (CI90.034-0.092); SRMR = 0.014]. Its explanatory power is moderate. The other variables in the model explain 36.4% of the variability of the scores in antisocial behavior.

**FIGURE 2:**
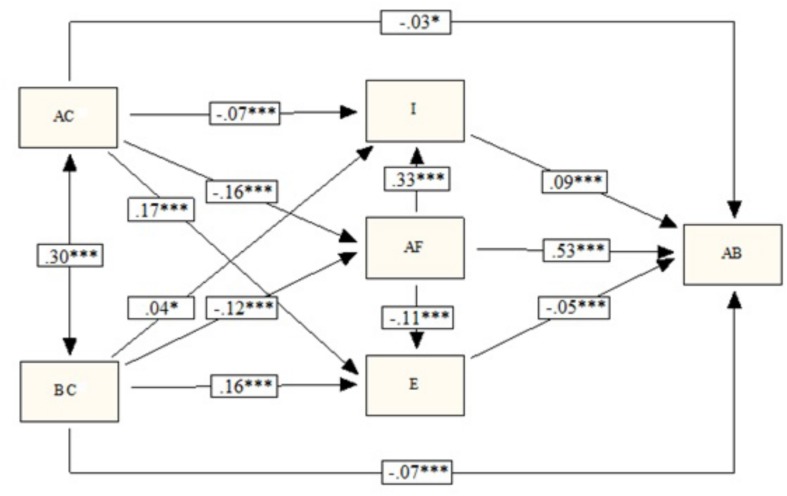
Result of path analysis (AC, affection and communication; BC, behavioral control; I, impulsivity; AF, antisocial friendships; E, empathy; AB, antisocial behavior). ^∗^*p* < 0.05; ^∗∗∗^*p* < 0.001.

As Figure 2 and Table 2 show, all of the effects were statistically significant, and were positive and negative in accordance with our hypothesis (Figure 1), except for the effect of behavioral control on impulsivity. Having antisocial friendships was positively related with engaging in antisocial behaviors. The effect of antisocial friendships is direct, and also indirect through its negative relationship with empathy, and positive relationship with impulsivity. The two analyzed dimensions of parenting style (affection and communication, and behavioral control) were positively correlated with each other. Both were negatively related to antisocial behavior. This effect is direct, and indirect through the positive relationship with empathy and the negative relationship with antisocial friendships. Impulsivity has a different mediating role with each of the two analyzed dimensions of parenting style. Affection and communication is negatively related to impulsivity, reducing the likelihood of antisocial behavior. In contrast, behavioral control is positively related to impulsivity, increasing the likelihood of antisocial behavior. The effect sizes are small or very small, except for the effect of antisocial friendships on impulsivity and on adolescent antisocial behavior, which is moderate.

**TABLE 2:** Regression weights of the model.

**Path**	**SRW**	**URW**	**SE**	**CR**	***p***	**Hypothesis testing**
AC BC	0.299^1^	2.307^2^	0.144	15.965	<0.001	Supported
AC AB	–0.032	–0.010	0.005	–2.050	0.040	Supported
AC I	–0.073	–0.092	0.023	–4.051	<0.001	Supported
AC AF	–0.164	–0.081	0.009	–8.975	<0.001	Supported
AC E	0.174	0.186	0.020	9.508	<0.001	Supported
BC I	0.041	0.051	0.022	2.289	0.022	Not supported
BC AF	–0.115	–0.055	0.009	–6.271	<0.001	Supported
BC E	0.155	0.163	0.019	8.548	<0.001	Supported
BC AB	–0.068	–0.021	0.005	–4.423	<0.001	Supported
AF I	0.331	0.856	0.045	19.187	<0.001	Supported
AF E	–0.113	–0.248	0.038	–6.462	<0.001	Supported
I AB	0.087	0.021	0.004	5.713	<0.001	Supported
AF AB	0.532	0.339	0.010	34.392	<0.001	Supported
E AB	–0.052	–0.015	0.004	–3.412	<0.001	Supported

*AC, Affection and communication; BC, Behavioral control; I, Impulsivity; AF, Antisocial friendships; E, Empathy; AB, Antisocial Behavior. SRW, Standardized Regression Weights; URW, Unstandardized Regression Weights; SE, Standard Error; CR, Critical Ratio. ^1^Correlation;^∼2^Covariance.*

The values in Figure 2 and Table 2 refer to the direct effects between the model variables. Table 3 shows the direct, indirect and total effects of each predictor variable on antisocial behavior. The overall effect of affection and communication on antisocial behavior is small, negative, and mainly indirect via empathy, antisocial friendships, and impulsivity, in order of magnitude. The overall effect of parental behavioral control on adolescent antisocial behavior is small, negative and mediated by empathy, antisocial friendships and impulsivity, in order of magnitude. In this case half of the overall effect is direct, and half indirect. The overall effect of antisocial friendships on adolescent antisocial behavior is moderate, positive and mainly direct. The indirect effect of antisocial friendships, mediated by impulsivity and empathy, is very small.

**TABLE 3:** Summary of standardized direct, indirect and total effects on antisocial behavior.

	**Effects**		
**Variables**	**Direct**	**Indirect**	**Total**
Affection and communication	–0.032	–0.108	–0.140
Behavioral control	–0.068	–0.070	–0.137
Antisocial friendships	0.532	0.035	0.567
Impulsivity	0.087	−	0.087
Empathy	–0.052	−	–0.052

## Discussion

The objective of this study was to analyze the effect of family (affection and communication, behavioral control) and friends (antisocial friendships) on antisocial behavior in adolescence, looking at both direct and indirect effects via adolescents’ impulsivity and empathy. The results are in line with the starting model (Figure 1), with the exception of the effect of behavioral control on impulsivity, which was the opposite of what we expected.

Parental affection and communication with their children have a protective effect, albeit small, on adolescent antisocial behavior. In line with previous research, affection and communication have a direct effect on antisocial behavior, but mostly the effect is indirect through the protective effect on antisocial friendships, and the adolescent’s impulsivity and low empathy (Brown et al., 1993; Wills et al., 2004; Hoskins, 2014; Boele et al., 2019).

Parental behavioral control of children generally has a protective effect, albeit small, on adolescent antisocial behavior. Again, in line with previous research, behavioral control has both direct and indirect effects on antisocial behavior through its protective effect on antisocial friendships and low empathy (Hoskins, 2014; Cutrín et al., 2015; Asano et al., 2016). However, contrary to our expectations, in this study we found that behavioral control can be a risk factor for antisocial behavior through its relationship with adolescent impulsivity.

Previous studies have found parental behavioral control to be positively related to self-control in their children (Li et al., 2015) and therefore negatively related to impulsivity. One possible explanation for that is that parental restrictions and monitoring may increase their children’s awareness of appropriate behavior, which may contribute to them learning to control their own behavior (Villanueva and Serrano, 2019). In our study, however, parental control was positively related to impulsivity in adolescent children, and thus with antisocial behavior. One explanation for this result may be that impulsive adolescents present more behavioral problems (Maneiro et al., 2017), and so may be subject to more control from their parents. In turn, these impulsive adolescents may respond negatively to behavioral control, and increase their impulsive, antisocial behavior. Another possible explanation is that in our study we examined the effect of each parenting style dimension separately (affection and communication; and behavioral control) rather than the combined effect of the two dimensions. Previous research has indicated that parental behavioral control has a positive effect on the prevention of antisocial behavior mainly when it occurs in a context of parental affection and communication, whereas hostile and intrusive control is a risk factor (Pinquart, 2017). Previous studies have also found an authoritarian parenting style, characterized by little affection and high control, to be a risk factor for impulsivity in adolescence (Li et al., 2018). This may be because authoritarian parents tend to be impulsive (Cox et al., 2018) and impulsivity is transmitted from parents to children (Higgins, 2009). Some researchers underline the importance of genetics in this transmission (Beaver et al., 2010), whereas others place more importance on education. Parents’ behavior is a model for their children. In addition, impulsive parents may generate an ineffective educational pattern which provokes anxiety and impulsivity in their children. They may be impatient with their children, less talkative and more inconsistent; they may generate hostile family environments; they may even consider certain inappropriate behavior in their children normal and appropriate (Meldrum et al., 2015).

Antisocial friendships are a risk factor for antisocial behavior in adolescence, with a moderate effect size. The effect is mainly direct, although as previous studies have indicated, there is also an indirect effect due to it being a risk factor for impulsivity and low empathy in adolescents (Burt et al., 2006; Padilla-Walker and Bean, 2009).

The results of this study have various practical implications. Firstly, all of the variables in the model are significant predictors of antisocial behavior in adolescence. This means that they should be borne in mind for the prevention and treatment of antisocial behavior in people of this age. Secondly, the effect of antisocial friendships in adolescence is greater than and contrary to the effects of the two analyzed parenting style dimensions (affection and communication, and behavioral control). Friendships may enhance or weaken parenting practices. The peer group can often encourage or approve of risky behaviors more than families (Sasson and Mesch, 2014; Shin and Ismail, 2014). Despite parents placing appropriate restrictions, occasionally peer pressure can lead adolescents to ignore them and engage in inappropriate behaviors. It is therefore important to teach adolescents to deal with peer and group pressure. Thirdly, one key variable in the prevention of adolescent antisocial behavior is the influence of the family on their children’s friendships. Although adolescents have increasing autonomy in choosing their friends, it depends to a large extent on the values transmitted by their parents. These values are largely communicated through the rules and limits on behavior set by parents and by the day-to-day family atmosphere, which serves as a model for behavior. Finally, impulsivity and low empathy are risk factors for antisocial behavior in adolescence. In the family and school context, therefore, social skills such as self-control and empathy should be encouraged (Díaz-Lopez et al., 2019).

This research contributes to the field of study, with significant practical implications. However, it is not without limitations. Firstly, the study was performed with a broad, random sample of adolescents, but constrained to some ages and a specific geographical location. Previous research has shown that exposure to the risk factors we examined, and their impact on the person, vary according to age and culture, which could also change their predictive power (Dekovic et al., 2004; Van der Put et al., 2011). For that reason, any generalization from these results to other ages or contexts should be made with caution. In the future, it would be interesting to replicate this research with other ages and in other contexts. Secondly, this study was specifically centered on the effect of family and friends on adolescent antisocial behavior, and the mediating role of adolescents’ impulsivity and empathy. However, previous research suggests that some of these associations are bidirectional (Salihovic et al., 2012; Pinquart, 2017). Future research should enhance the starting theoretical model in order to improve its predictive capacity. Thirdly, the conclusions are limited by the variables included in the model. Although the variables we included were relevant, there are other variables that might interact with those included in this study and influence the likelihood of an adolescent engaging in antisocial behavior (Assink et al., 2015). Fourth and lastly, this was a cross-sectional study. It would be interesting to test whether the hypothesized causal relationships would be confirmed in a longitudinal study. Despite these limitations, this study is a contribution to clarifying the causal mechanisms of adolescent antisocial behavior, as a basis for its prevention and treatment.

## Data Availability

The datasets generated for this study are available on request to the corresponding authors.

## Ethics Statement

This study was carried out in accordance with the recommendations of the Deontology Commission of the General Counsel of Psychology of Spain, with written informed consent from all subjects. All subjects gave written informed consent in accordance with the Declaration of Helsinki. The protocol was approved by the research and ethics committee at the University of Oviedo.

## Author Contributions

DA-G and JN designed the study, analyzed the data and drafted the manuscript. PG-C, CR, and RC critically reviewed the draft and made significant contributions to the final version.

## Conflict of Interest Statement

The authors declare that the research was conducted in the absence of any commercial or financial relationships that could be construed as a potential conflict of interest.
